# COVID-19 testing, infection rates, and related outcomes in adults with intellectual and developmental disabilities (IDD): An application of linked administrative health data to support Ontario’s COVID-19 response.

**DOI:** 10.23889/ijpds.v7i3.2025

**Published:** 2022-08-25

**Authors:** Natalie Troke, Diana An, Eliane Kim, Yona Lunsky, Robert Balogh, Lesley Plumptre

**Affiliations:** 1 ICES; 2 CAMH; 3 Ontario Tech University

## Objectives

People with intellectual and developmental disabilities (IDD) have been disproportionately impacted by COVID-19, a population more likely to experience poor health outcomes. An Applied Health Research Question (AHRQ) request through ICES led to an investigation that monitored COVID-19 related infections and outcomes in adults with and without IDD.

## Approach

The ICES-AHRQ team approved a request from the Ministry of Children, Community and Social Services (MCCSS), to determine the proportion of adults with IDD tested and confirmed positive for COVID-19. The scientists also explored if positive IDD cases experienced similar health outcomes to the general population. The open cohort was derived by linking those with a COVID-19 test in Ontario Laboratories Information System, from January 2020 to December 2021, to supplemented positive case data and administrative health databases. An algorithm was used to define IDD by a series of inpatient hospitalizations, emergency department and/or physician visits, with IDD diagnostic codes.

## Results

Similar rates of testing and positivity were observed for those with (46%; 6%) and without IDD (43%; 5%). However, adults with IDD confirmed positive for COVID-19 were mostly male (62% vs 49%), aged 18-29 (40% vs 27%), had medical conditions associated with frailty (15% vs 4%), and from the lowest neighborhood income quintile (27% vs 23%), compared to the non-IDD population, respectively. Cumulatively, deaths and hospitalizations following a COVID-19 diagnosis were two-times more likely in the IDD population, while comparable rates of intensive care unit (ICU) admissions were reported. Particularly, adults with IDD aged 18-54 experienced higher rates of hospitalizations (48% vs 30%), ICU admissions (56% vs 36%), and deaths (26% vs 6%), compared to the same age group in those without IDD.

## Conclusion

Real-time administrative health data and analytics were used to support Ontario’s COVID-19 response in those with IDD. To inform decision making and policy actions related to immediate testing strategies, MCCSS used such findings to increase the availability of testing for adults with IDD, who may face multiple barriers.

